# Virtual reality in managing dental pain and anxiety: a comprehensive review

**DOI:** 10.3389/fmed.2023.1285142

**Published:** 2023-12-05

**Authors:** Lin Fan, Jie Zeng, Longkuan Ran, Chao Zhang, Jing Wang, Cong Yu, Nan Zhao

**Affiliations:** ^1^Department of Anesthesiology, Stomatological Hospital of Chongqing Medical University, Chongqing, China; ^2^Chongqing Key Laboratory of Oral Diseases and Biomedical Sciences, Chongqing, China; ^3^Chongqing Municipal Key Laboratory of Oral Biomedical Engineering of Higher Education, Chongqing, China

**Keywords:** virtual reality, dental treatment, dental anxiety, pain, behavior management, distraction therapy

## Abstract

**Objectives:**

This study aimed to identify, analyze, and summarize the clinical efficacy of virtual reality (VR) distraction therapy for oral treatment in different hospital settings in contrast to medical interventions that induce anxiety and pain. Furthermore, this review aimed to determine the implications for research and clinical practice of VR distraction therapy.

**Data:**

This review investigated the clinical efficacy of VR in the oral treatment of procedural pain or anxiety. Quality assessment of the included studies was conducted. A narrative synthesis of the collected data was performed.

**Sources:**

Literature studies from six electronic databases were searched for a comprehensive review, namely, the Cochrane Oral Health’s Trials Register, Cochrane Central Register of Controlled Trials (Central), MEDLINE (PubMed), EMBASE, Scopus, and Web of Science.

**Study selection:**

One thousand five hundred twenty-two patients aged between 0 and 60 years who used VR during dental treatment were included in this review. Among these studies, 8 and 14 studies comprised adult and pediatric patients.

**Conclusion:**

Overall, the reviewed studies underscore the efficacy of VR to mitigate pain and anxiety in the context of dental treatment. VR is an innovative pain and anxiety management approach that facilitates dental treatment patients to immerse themselves in a virtual world while using distractions to reduce pain and anxiety.

**Clinical significance:**

VR is an effective and novel non-pharmacological method of behavioral management that contributes to improving medication safety for dental patients. VR as a distractive approach can reduce the fear associated with medical interventions and prevent severe pain sensitivity, anxiety, and medical avoidance among adults and children.

## 1 Introduction

Patients visiting dental treatment clinics often experience pain or anxiety before or during treatment. Fear-related behaviors, which can disrupt good dental procedures, have been considered the most challenging aspect of treatment avoidance among patients (1). Many patients with such fear visit the dentist only when they are in pain, increasing the likelihood of experiencing further pain (2). This, in turn, can increase pain sensitivity and heighten fear, anxiety, and medical avoidance among patients (3). A study found that dental anxiety ranked fifth among the most common fearful conditions, although its prevalence decreased with increasing age (4). Consequently, pain associated with medical practice remains a frequent problem for pediatric patients (5). Clinical pain and anxiety relief vary in terms of the level of interaction and engagement, ranging from passive to active distraction patterns, with active distraction consuming more attentional resources (2).

Currently, oral health practitioners have numerous approaches for reducing patient anxiety and pain caused by oral treatment. Dental anxiety has been a common challenge among dental care providers who use traditional behavioral management techniques to reduce dental anxiety, including narration, desensitization, voice control or hypnosis, applied behavior analysis, positive reinforcement, distraction, and parental presence or absence (6, 7). Studies have shown that patients with high levels of trait anxiety typically report higher levels of anxiety and pain during dental procedures (8). In addition to cognitive behavioral management, doctors often use drug management methods. Following the treatment gradient, preoperative anti-anxiety drugs, treatment process sedation (laughing gas and conscious sedation), and general anesthesia are administered (9–11).

Another non-pharmaceutical technology that is gaining the attention of dental practitioners is virtual reality (VR). VR technology creates a highly realistic three-dimensional (3D) virtual environment to help patients escape the real world through a variety of sensory stimuli, such as visual, auditory, tactile, and olfactory stimuli (12). By stimulating visual, auditory, and proprioceptive senses, VR can act as a distraction that interferes with the processing of noxious stimuli in patients (13). Virtual reality technology has been applied in a wide range of fields; in medicine, it is being applied in the fields of rehabilitation and clinical medicine (14). In obstetric labor pain, considered to be the limit of human pain, studies have shown that chewing gum and virtual reality are easy to use and effective during labor as painless anti-anxiety methods (15). Several studies have suggested that immersive VR may serve as a viable non-pharmacological analgesia (16–18), while another reported that VR, as a non-pharmacological option, may be more effective than traditional analgesia (19). Moreover, VR may influence the extent of opioid abuse, serving as an advantage for opioid-dependent patients (20). Patients preferred VR distraction methods during treatment, in contrast to groups without VR condition and movie viewing (21). Owing to its inherent immersive, imaginative, and interactive nature, VR is suitable for non-pharmacological behavioral management during dental treatment.

VR distraction techniques have been used to reduce pain during burn wound care (2). Furthermore, it has been hypothesized that VR experiences can overcome pain by consuming an individual’s limited cognitive attentional resources. Thus, the pain experienced is reduced by shifting patients from painful stimuli to a pleasant virtual world (22). These findings present the first evidence for VR efficacy, which has driven many subsequent studies. Meanwhile, more attention should be paid to adverse reactions when patients use VR. A few patients have reported adverse reactions, such as nausea and headache, after using VR (19, 21). Exposure to the VR environment may cause cybersickness, with symptoms including nausea, dizziness, headache, blurred vision, and a feeling of moving through space. Currently, we summarize and compare the findings of using VR to mitigate pain and anxiety and its possible adverse reactions in dental treatment healthcare settings.

### 1.1 Objectives

This review study aimed to systematically review, evaluate, and summarize the results of studies investigating the impact of pain and anxiety among patients undergoing different surgical modalities using VR distractions throughout the perioperative period of dental treatment in medical settings. Furthermore, the implications of VR for research and clinical practice are considered.

## 2 Materials and methods

### 2.1 Study design

Following the Preferred Reporting Items for Systematic Evaluation and Meta-Analysis (PRISMA) guidelines and checklist (23), a comprehensive evaluation design was prepared to systematically review, assess, extract, and summarize the available data on the clinical efficacy of VR distractions for procedural pain and anxiety. The methodological rigor of the evaluation is similar to that of a systematic review; however, the former enables the inclusion of quantitative, qualitative, mixed-methods studies, and case reports (24).

### 2.2 Information sources and search strategies

Research articles published between 2000 and 2023 were searched using the following six electronic databases: Cochrane Oral Health’s Trials Register, Cochrane Central Register of Controlled Trials (Central), MEDLINE (PubMed), EMBASE, Scopus, and Web of Science. The search terms included virtual reality, dental treatment, dental surgery, procedural pain, procedural anxiety, pain control, anxiety control, dental anxiety, and related keywords. The search was limited to studies published in English and with samples comprising adults and children (aged 0–60 years). No attempts were made to locate or contact researchers for unpublished studies.

### 2.3 Study selection

Study titles and abstracts were screened for inclusion by a reviewer. In cases of uncertainty, reviewers consulted with members of the research team, who discussed collaboratively until a consensus was reached.

### 2.4 Eligibility criteria

#### 2.4.1 Participant characteristics

Studies with samples that included patients aged 0–60 years who underwent dental treatment involving VR distraction were considered in this review.

#### 2.4.2 Types of outcome measures

Studies designed to investigate the use of VR distraction in the management of procedural pain or anxiety during the perioperative period of dental treatment were included.

#### 2.4.3 Types of research

Quantitative, qualitative, and mixed-methods studies as well as case reports were reviewed. There was no minimum threshold imposed for the quality assessment scores.

#### 2.4.4 Review methodology

The reviewers screened the titles and abstracts for eligibility. Full-text articles of the selected titles and abstracts were sought. Thereafter, the full-text articles were read by one reviewer to determine their eligibility for inclusion. The references of the selected full-text articles were scanned to identify additional relevant studies, and reviewers sought assistance from the research team when they were uncertain of an article’s eligibility for inclusion.

### 2.5 Data extraction

The studies considered for review were first categorized according to the type of medical procedure stipulated in the end notes. Thereafter, data were extracted and inserted into a table using Microsoft Word. Separate tables were created for each medical procedure group. One reviewer performed data extraction, while another validated the extracted data for each study based on the authors, study design, procedures, sample characteristics, and pain and anxiety outcomes.

### 2.6 Quality assessment

A reviewer assessed the studies using the Mixed Methods Appraisal Tool (MMAT) (25) and Joanna Briggs Institute (JBI) Critical Appraisal Checklist (26). The MMAT was designed for the quality assessment phase of a systematic review of mixed-methods studies, including qualitative studies, randomized and non-randomized controlled trials, quantitative descriptions, and mixed-methods studies. Each study was assessed using five criteria, with scores ranging from 0% (criteria not met) to 100% (all criteria met). The JBI Critical Appraisal Checklist is a peer-reviewed assessment tool used for case reports in a comprehensive evaluation. Each case report was assessed based on eight questions, with scores ranging from 0% (questions not answered) to 100% (all questions answered). Despite the quality assessment scores, all studies and case reports were retained for analysis.

### 2.7 Data analysis

A descriptive analysis of the sample and study characteristics was performed on the data extracted into tables to generate the MMAT and JBI quality scores using the constant comparison method described by Whittemore and Knafl (24). For ease of analysis, the data were first categorized into medical procedure subgroups and further divided into VR interventions and devices, then pain and anxiety outcome subgroups. The data were organized in tables and graphs that highlight the similarities and differences identified through an iterative process. Finally, after comparing the findings and considering confounding variables, generalized conclusions were drawn and presented as themes.

## 3 Results

### 3.1 Search results

**Figure 1:**
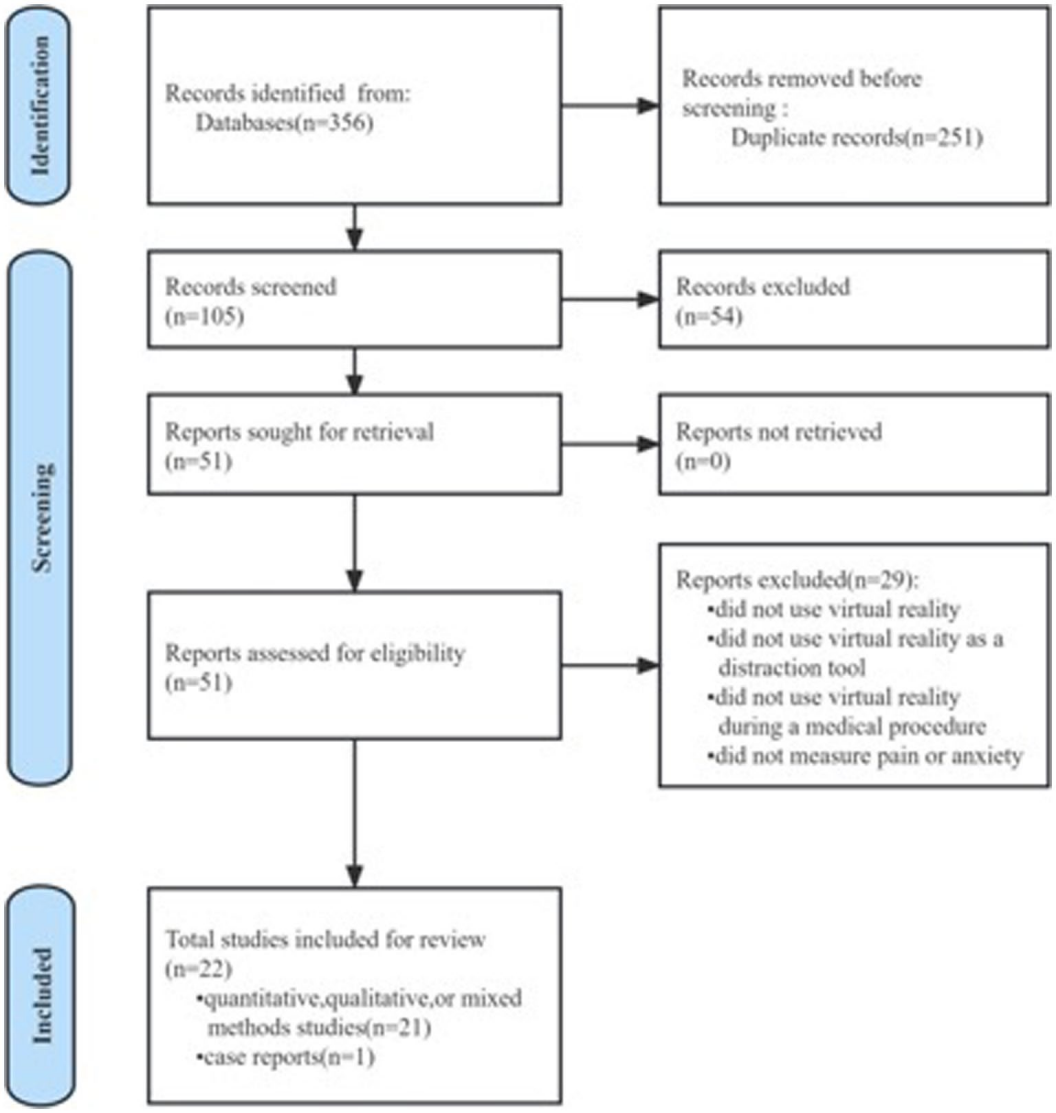
PRISMA flow diagram of study selection.

Overall, 356 articles were related to dentistry, VR, pain, and anxiety. After removing duplicates, 102 titles and abstracts and 51 full-text articles were screened for eligibility. Following the screening, 22 studies (2, 4, 19, 21, 27–44) were included in the final review (Figure 1).

### 3.2 Study and sample characteristics

A total of 22 eligible studies were published between 2000 and 2023. These studies varied in design and comprised intra-subject randomized controlled trials (*n* = 6) (28, 29, 31, 36, 40, 41), inter-subject randomized controlled trials (*n* = 13) (2, 4, 19, 21, 27, 32–35, 38, 42–44), randomized single-blind controlled crossover modalities (*n* = 2) (30, 37), and mixed-methods randomized controlled trials (n = 1) (39). Notably, 1,522 dental treatment patients aged 0–60 years underwent VR interventions with the following procedures: periodontal scaling and root planning (*n* = 2) (19, 21), periodontal treatment (*n* = 1) (2), local anesthetic procedures (*n* = 3) (4, 28, 32), mandibular blocked third molar extraction (*n* = 2) (36, 41), dental surgery (*n* = 6) (19, 27, 30, 31, 39, 40), dental treatment (*n* = 2) (33, 43), preoperative intervention (*n* = 1) (35), endodontic treatment (*n* = 2) (37, 38), and deciduous tooth extraction (*n* = 3) (34, 42, 44).

#### 3.2.1 Virtual reality software used

Various VR interventions were delivered, including in the degree of interaction (Table 1). Some studies used a VR interactive game, such as SnowWorld or Undersea Landscape (*n* = 3). In other studies, patients experienced an adventure during their dental treatment (*n* = 6). The majority of studies used VR watching a video or images (*n* = 13).

**Table 1:** Virtual reality intervention used in 22 eligible studies conducted in dental treatment.

VR intervention	Interactive game	3D experience/adventure	Watching a video or images
Adult	*n* = 1	*n* = 4	*n* = 3
Child	*n* = 2	*n* = 2	*n* = 10
Total	*n* = 3	*n* = 6	*n* = 13

#### 3.2.2 Virtual reality in adult dental surgery

Table 2 presents eight of the 22 studies that investigated the use of VR distraction in dental treatment among 615 adult patients.

**Table 2:** Studies in adults using virtual reality during oral therapy: characteristics and findings.

Study authors	Design	Procedures	Sample characteristics	Sample age	VR headset	VR intervention	Pain	Anxiety
Hoffman et al. (2)	-Inter-subject randomized control -Conventional + watch movies + VR	-Periodontal treatment	*n* = 2	Age range = 50–60 years	V8 VR helmet Virtual Research Systems，Santa Clara，California	Interactive game	Subjective pain ratings - Patient 1 had no distraction (7.2), watching movies (7.2), VR (1.2) -Patient 2 had no distraction (4.4), watching movies (3.3), VR (0.6)	
Furman et al. (21)	-split-mouth design -Inter-subject randomized control -Conventional + watch movies + VR	-Periodontal scaling and root planning	*n* = 38 Men = 17 Women = 21	Age range = 30–60 years Average age = 45.9 ± 12.6 years	V8 VR helmet Virtual Research Systems，Santa Clara，California	3D experience/adventure	- Significantly lower mean VAS pain score VR was (1.76, SD 1.4) Movies was (2.57, SD 1.8) The control condition was (3.95, SD 2.1)	
Alshatrat et al. (19)	Within-subject/split-mouth design -Conventional + VR	Periodontal scaling and root planning	*n* = 50 Men = 22 Women = 28	Age range = 18–54 years Average age = 36 ± 11.843 years	Composed of a binocular head‐mounted display (iWear Video Headphones; Vuzix^®^, Rochester, NY, United States) by Personal Computer (DELL^®^, Round Rock, TX, United States)	3D experience/adventure	- The VAS scores for average pain perception were significantly lower during VR (mean rank = 11.05) compared to without VR (mean rank = 10.00).	
Sweta et al. (4)	Inter-subject randomized control Conventional + VR	Local anesthesia	*n* = 50	Age range = 24–56 years Average age = 39.72 ± 15.93 years	VR through the use of a head-mounted immersive type of display powered by a smartphone.	Watching a video or images	The VAS pain scores of the patients were statistically analyzed. The postoperative VAS pain score was 2.60 ± 1.384 in the control group and 1.28 ± 0.891 in the study group.	
Yamashita et al. (41)	Inter-subject randomized control Conventional + VR	Extraction of an obstructed third molar in the lower jaw	*n* = 100	Age range = adult	Company PR NETWORK Co. (Fukuoka, Japan) for the purposes of relaxation.	Watching a video or images		The difference in anxiety measured using the VAS before and during treatment for the VR group coke was −13.3 ± 28.7 mm. Control patients had an increase of 4.0 ± 22.3 mm.
Lahti et al. (35)	Inter-subject randomized control Conventional + VR	Pre-operative dental intervention	*n* = 280	Age range = adult	Samsung Gear VR headset	3D experience/adventure		Dental anxiety decreased more in the VR group than the TAU group (*β* = −0.75, *p* < 0.001, for MDAS total score; *β* = −0.43, *p* < 0.001, for anticipatory anxiety score) in patients of a primary Dental care clinic. In women, dental anxiety decreased more in VRR than TAU for total MDAS score (*β* = −1.08, *p* < 0.001) and treatment-related dental anxiety (*β* = −0.597, *p* = 0.011). Anticipatory dental anxiety decreased more in VRR than TAU in both men (*β* = −0.217, *p* < 0.026) and women (*β* = −0.498, *p* < 0.001).
Mladenovic and Djordjevic (36)	Inter-subject randomized control Conventional + VR	Extraction of an obstructed third mandibular molar	*n* = 74 Men = 46 Women = 28	Age range = 20–54 years Average age = 34.9 ± 9.0 years	Sam sung Gear VR Oculus and Samsung Galaxy S10 − Android	3D experience/adventure	During surgery with VR goggles, respondents experienced significantly lower pain values during anesthesia application and surgical extraction of the third molar.	
Almugait and AbuMostafa (28)	Inter-subject randomized control Conventional + VR	Local anesthesia	*n* = 21 Men = 10 Women = 11	Age range = 25–60 years Men average age = 34.5 years Women average age = 35.4 years	128 GB Oculus Quest^®^ (Facebook Inc., United States)	Watching a video or images	The difference between virtual reality in reducing pain caused by dental injections was statistically insignificant. The effect was comparable to that of local anesthesia.	

#### 3.2.3 Virtual reality in pediatric dental surgery

Table 3 presents 14 of the 22 studies that investigated the use of VR distraction in dental treatment among 907 pediatric patients.

**Table 3:** Studies on the use of virtual reality in oral therapy in children: characteristics and findings.

Study authors	Design	Procedures	Sample characteristics	Sample age	VR headset	VR intervention	Pain	Anxiety
Aminabadi et al. (30)	Randomized single-blind controlled crossover approach Conventional + VR	Dental surgery	*n* = 120 Boys = 63 Girls = 54	Age range = 4–6 years Group 1 average age = 5.18 ± 0.67 years Group 2 average age = 5.65 ± 0.71 years	i-glasses 920HR Ilixco, Inc., Menlo Park，CA, United States	Watching a video or images	Facial pain scores: in group 1, the scores during the first (with VR distraction) and second (without VR distraction) treatments were 1.89 ± 0.65 and 3.00 ± 0.81, respectively and there was a statistically significant increase. In group 2, the mean of the scores was 3.05 ± 0.60 for the first (without VR distraction) treatment and 2.05 ± 0.60 for the second (with VR distraction) treatment pain scores	MCDAS(f) anxiety score: in group 1, the mean in the first (with VR distraction) and second (without VR distraction) sessions were 12.58 ± 1.01 and 17.68 ± 1.25, respectively. Statistically significant increase. In group 2, the mean MCDAS(f) anxiety score was 18.25 ± 1.02 in the first session (without VR distraction) and 13.20 ± 1.00 in the second session (with VR distraction).
Panda (38)	Inter-subject randomized control Conventional + VR	Inferior alveolar nerve block -Endodontic treatment of mandibular deciduous molars	*n* = 30	Age range = 6–8 years	Epson Moverio BT-100	Watching a video or images	Faces Pain Scale Revised (FPS-R). 1.73 and 5.73 in the VR group and control group, respectively.	
Atzori et al. (31)	Using a within-subjects design Conventional + VR	Dental surgery	*n* = 5 Boys = 3 Girls = 2	Age range = 7–17 years Average age = 13.20 ± 2.39 years	Oculus Rift VR	Interactive game	“Pain discomfort”: mean without virtual reality was 2.40 (SE = 1.52), dropping to 0.60 (SD = 0.55) with virtual reality, *t*(4) = 3.67, *p* < 0.05, SD = 1.10. “Worst pain”: mean without virtual reality was 3.80 (SD = 2.59), mean with virtual reality decreased to 2.20 (SD = 1.79), *t*(4) = 3.14, *p* < 0.05, SD = 1.14.	
Niharika et al. (37)	Randomized single-blind controlled crossover approach -Conventional + VR	Local anesthesia Endodontic treatment of deciduous teeth	*n* = 40 Boys = 22 Girls = 18	Age range = 4–8 years Group 1 average age = 7.17 ± 0.316 years Group 2 average age = 7.28 ± 0.300 years	Google VR Box and Anti-Tank Virtual Reality 3D Glasses	Watching a video or images	In group 1, the mean facial pain scores during the second (with VR retraction) and third (without VR retraction) sessions were 2.56 ± 0.390 and 5.22 ± 0.515, respectively, which was a statistically significant increase. In group 2, the mean facial pain score during the first session (without VR retraction) was 5.44 ± 0.682 and the mean pain score during the second session (with VR retraction) was 2.33 ± 0.370.	In group 1, the mean MCDAS (f) anxiety scores in the first (with VR distraction) and second (without VR distraction) sessions were 14.72 ± 0.843 and 19.38 ± 0.897, respectively. These values indicate a statistically significant increase in anxiety scores. In group 2, the mean MCDAS(f) anxiety score was 19.56 ± 0.883 for the first session (without VR distraction) and decreased to 14.44 ± 0.805 for the second session (with VR distraction).
Shetty et al. (40)	Inter-subject randomized control Conventional + VR	Dental surgery	*n* = 120 The distribution of girls and boys was the same in the control and study groups	Age range = 5–8 years	i-glasses 920HR, Ilixco Inc.	Watching a video or images		Mean and median anxiety scores Before treatment. 6.18 (3.84) and 15 (14,17.75), 16.82 (3.80), and 17 (14,19) for the VR and control groups, respectively After treatment. 11.28 (3.51) and 10.5 (9,13), 16.47 (3.48), and 16 (14,18) in the VR and control groups, respectively.
Koticha et al. (34)	The split-mouth study design. Conventional + VR	Extraction of deciduous teeth	*n* = 30	Age range = 6–10 years	BlackBug Virtual Reality Glasses 3D VR Box headsets for 4.7–6 inch mobile phones, model no: a236, India	Watching a video or images		Mean Venham picture test values Before extraction. 2.9667 ± 0.2815 and 3.60 ± 0.327 for the VR and control groups, respectively After tooth extraction. 3.50 ± 0.2815 and 3.80 ± 0.353 for the VR and control groups, respectively
Alshatrat et al. (29)	Inter-subject randomized control Conventional + VR	Dental surgery	*n* = 54 Boys = 22 Girls = 32	Age range = 6–11 years Average age = 8.39 ± 2.05 years	iWear Video Headphones (Vuzix^®^).	Watching a video or images	During VR, total scores on the VAS Most Severe Pain, VAS Discomfort, Wong–Baker FACES Scale, and FLACC scale were statistically lower than total scores in the absence of VR.	
Ran et al. (39)	Mixed randomized control Conventional + VR	Dental surgery	*n* = 120 Boys = 63 Girls = 57	Age range = 4–8 years VR group average age = 5.59 ± 0.92 years TSD Group average age = 5.66 ± 0.99 years	HTC (Hsinchu, Taiwan, China)‘s VIVE VR	Interactive game	Intraoperative pain scores VR group and control group were (1.58 ± 1.08), (2.86 ± 0.96)	Before and after CFSS-DS intervention. The VR group was 34.17 ± 5.81 and 24.77 ± 6.98. 34.08 ± 8.42 and 27.98 ± 7.41 for the TSD group.
Gómez-Polo et al. (33)	Inter-subject randomized control Conventional + VR	Dental treatment	*n* = 80 Boys = 35 Girls = 45	Age range = 5–10 years Average age = 7.9 ± 1.6 years	Carl Zeiss AG, Oberkochen Germany VR	Watching a video or images		According to the Corah test (CDAS), the majority of parents felt relaxed (46.3%) or slightly worried (28.8%) about their child’s dental treatment, although no differences were found between the control and VR groups. Furthermore, anxiety levels at the first appointment were comparable between the groups, with only 10% of children feeling sad or very sad. However, child behavior at baseline (at the first appointment) was significantly lower in the VR group (25% of children behaving negatively) than in the control group (10% of children behaving negatively)
Aditya et al. (27)	Inter-subject randomized control Fingertip Gyro + Kaleidoscope + VR + Conventional	Dental surgery	*n* = 60	Age range = 6–9 years	MI VR Headset， India	Watching a video or images		Self-reported anxiety for the four groups was measured using (Venham Picture Test VPT) Mean Score and S.D. were: 1.1111, 1.2472; 2.2667, 2.7086; 1.8444, 1.7832; 4.1111, 2.7898
Felemban et al. (32)	Inter-subject randomized control 2D Video + VR	Local anesthesia	*n* = 50 Boys = 21 Girls = 29	Age range = 6–12 years Average age = 8.4 ± 1.46 years	LG 360 virtual reality (VR) headset, LG Electronics	Watching a video or images	Regardless of the distraction technique used, mean FLACC Behavioral Pain Assessment Scale scores were higher in younger subjects and females (*p* = 0.034 and *p* = 0.004, respectively). Lower FACES Pain Rating Scale scores (2.40 ± 2.82) were not statistically significant (*p* = 0.497).	
Du et al. (42)	Inter-subject randomized control Conventional + VR	Extraction of deciduous teeth	*n* = 128 Boys = 68 Girls = 60	Age range:4.3–8.8 years Average age:6.3 ± 3.5 years	HTC Vividu Chengdu, China	3D experience/adventure	The Wong–Baker Scale score (3.47 ± 0.76) score in the VR group was significantly lower than that in the control group (5.56 ± 1.1 3, *p* = 0.015)	The CFSS-DS score in the VR group was significantly reduced after dental treatment (34.58 ± 6.90 before surgery and 32.32 ± 15.58 after surgery, *p* = 0.02)
Mehrotra and Manju (43)	Inter-subject randomized control Conventional + VR+ Audio	Dental treatment	*n* = 40	Age range:6–14 years	PROCUS ONE VR	3D experience/adventure		After the introduction of VR distraction technology, Venham anxiety scores can be successfully reduced in children with mild intellectual disabilities and healthy children undergoing dental restorative treatment.
Pathak et al. (44)	Inter-subject randomized control Conventional + VR	Extraction of deciduous teeth	*n* = 30 Boys = 15 Girls = 15	Age range:6–12 years	VR SHINECON	Watching a video or images		Through heart rate assessment, children can use virtual real-life equipment to reduce anxiety during tooth extraction during a molar.

#### 3.2.4 Virtual reality to alleviate pain associated with dental treatment

Fourteen studies (six and eight adult and pediatric studies, respectively) examined the effects of VR distraction on pain perception. These studies found that VR distraction significantly reduced pain perception in contrast to standard care (4, 19, 36, 38, 39, 42). Two studies (2, 21) also reported that VR distractions significantly reduced pain in contrast to standard care and movie viewing. Similarly, significant reductions in “worst pain” and “pain discomfort” were reported (31) during VR distraction, in contrast to using standard analgesia exclusively. Furthermore, a significant reduction in “worst pain” and “pain discomfort” was reported (29) among children undergoing dental surgery requiring local anesthesia (30, 31). Moreover, studies using a randomized single-blind controlled crossover approach, applying within-group comparisons, found that pain scores were controlled and decreased when VR distraction was used.

Three studies (4, 37, 38) showed that VR distraction during local anesthesia was effective in reducing pain scores. In contrast, another study (32) showed an increased likelihood of higher pain scores during local anesthetic administration among female participants and younger groups, regardless of the distraction used. Another study (28) showed that the effect of VR on pain reduction caused by dental injections was statistically significant, similar to that of local anesthesia.

#### 3.2.5 Virtual reality reduces anxiety associated with dental treatment

Eight studies found that VR significantly reduced state anxiety (34, 35, 39–44). Two studies (30, 37) measured anxiety scores with and without VR distraction and found that VR distraction was effective in reducing the state of anxiety among the study group.

#### 3.2.6 Virtual reality improves other markers related to dental treatment

Several studies have underscored the impact of VR distraction on other markers in addition to pain and anxiety. One study (21) reported that in both VR and movie distraction conditions, patients demonstrated significantly lower vital signs during treatment as opposed to controls, as well as during VR in contrast to watching movies. However, a different study (34) found that VR significantly reduced physiological parameters associated with anxiety (pulse and oxygen saturation) despite contradictory self-reports from children. Another study (19) showed that participants had significantly lower systolic blood pressure after treatment with VR, whereas no differences were observed in diastolic blood pressure and pulse rate. Moreover, VR was found to have a significant reduction effect on physiological parameters (4). Similarly, a study (36) reported that when VR was used, participants demonstrated significantly lower heart rate values before and during the procedure. In contrast, a different study (32) found that the mean heart rate in the VR group was significantly higher than that in the control group (i.e., the audio-visual (AV)-viewing group) when compared with all time-points, excluding the baseline. Parasympathetic activity was shown to be slightly dominant in another study (41), whereas both sympathetic and parasympathetic activities were in equilibrium among patients during VR, indicating a shift toward a stable mental state. A significant decrease in salivary cortisol levels during short-term invasive dental treatment in children was reported using VR distractions (40). Finally, the use of VR distraction was shown (39) to shorten the duration of dental procedures and improve compliance among children undergoing short-term dental procedures.

#### 3.2.7 Adverse reactions during virtual reality use

The use of VR distraction in dental treatment for procedural pain and anxiety management has been widely acknowledged, despite reports of adverse effects. Five out of 38 study participants reportedly experienced mild nausea in the VR condition, but not while watching a movie (21). Hoffman et al. showed that 94% of participants did not feel nauseated when experiencing the virtual world (18). Aditya et al. (27) showed that more than 90% of respondents did not experience any VR-related discomfort during the procedure, while the remainder felt partial discomfort. Atzori et al. (31) showed that only two children were observed to be uncomfortable with VR distraction, requiring the removal of the device, and were excluded from the study.

## 4 Discussion

This comprehensive review systematically evaluated, extracted, and summarized the data from 22 studies that explored the use of VR distraction in the management of procedural pain and anxiety during dental treatment. VR has been tested and contrasted with the group without VR condition and movie viewing, demonstrating the effectiveness of its use as an innovative intervention to alleviate procedural pain and anxiety among dental treatment patients. This comprehensive review summarizes the available evidence on the use of VR during dental treatment, describes the clinical efficacy outcomes, and subsequently considers the implications for research and clinical practice.

### 4.1 Virtual reality and procedural medical practices resulting in pain

The studies included in this review support the analgesic effects of VR in oral treatment procedures. Among the factors hypothesized to contribute to VR’s analgesic effects include the degree of immersion and level of interaction (2, 14). Several studies have shown that a high-quality VR headset can block visual and auditory pain cues present in the clinical setting, thus contributing to pain relief and enhancing the sense of presence in the virtual world (4, 19, 22, 38, 39, 45). One study (4) showed that distraction interventions and hypnosis techniques can be used to treat pain. These distraction interventions are preferred by patients because they are non-invasive and non-pharmacological. The immersive nature of VR has been shown to distract children’s attention, thus manipulating pain perception and reducing the intensity of pain (30, 46). Furthermore, a significant 42% reduction in “worst pain score” and a 75% reduction in “pain discomfort” scores were reported among patients during VR (31). The greatest total analgesic effect may be achieved by combining immersive interactive VR with traditional pain medications (46). A review study (22) revealed that VR for pain management is effective in reducing pain during dental treatment in both children and adults; however, it has a greater potential for children.

#### 4.1.1 Virtual reality and anxiety arising in medical practice

Previous literature supports the use of distraction to manage procedural anxiety (22, 47), which is consistent with the findings of this review. This review finds that VR is an effective method for anxiety relief (30, 34, 35, 37, 39–41, 48). Among these, only two studies (35, 41) have underscored the efficacy of VR distraction among adults. One study (30) reported that the positive effects of VR distraction on pain and anxiety in children were attributed to the complete blockage of the child’s visual field as well as to successful distraction techniques. These benefits may be related to the increasingly immersive images that result from the combination of audio, visual, and kinesthetic sensory modalities in VR. Furthermore, VR distraction is a pleasant experience that can reduce negative emotions, such as anxiety, by removing the user from medical situations that would trigger anxiety (49). A study (37) reported that people with higher trait anxiety felt increased threat and responded poorly to distraction techniques compared to people with lower trait anxiety. However, VR distraction has greater potential to alleviate the pain and anxiety associated with various dental procedures. It is a safe, non-invasive technique that does not require prior education or training and has long-term effects in terms of creating more positive memories during treatment, thus increasing the willingness of patients to return for treatment.

#### 4.1.2 Virtual reality and adverse reactions in medical practice

In their review, Wismeijer et al. described “simulator sickness” as the result of proximity and lower quality images projected through the VR device. This was expected to cause nausea in sensitive individuals (50). Hoffman et al. suggested that longer exposure durations to VR are more likely to be a problem (2). Meanwhile, another study indicated that the incidence of cybersickness in the virtual environment varies depending not only on the length of exposure but also on the type of simulation and complexity of the devices (51). In their research, Weech et al. described that when the patient’s gaze follows the text or images moving rapidly on the screen, it results in headaches, nausea, and insecurity (52, 53). We summarized the suggestions for reducing VR-related adverse reactions as follows. Keep patients’ heads during exposure to VR. VR hardware and software should be designed to minimize simulator sickness, and individuals with high susceptibility to cybersickness likely should not be administered VR.

### 4.2 Study significance

VR distraction can be used as a successful behavioral modification method for children aged 5–8 years undergoing short-term invasive dental treatment (2). The benefits of VR include ease of use, improved treatment control, and safety for most patients. Instruction is not needed for either the patients or treatment staff. Moreover, the frequent use of the technology does not diminish its positive effects (54); thus, it can be used on children with minor adjustments to size. The most significant advantage of this behavior management technique is that it is equally acceptable for parents and children (50). Some studies have suggested that anxiety is related to several factors, including age, gender, type of dental treatment, parental anxiety, and socioeconomic status. These factors may influence the efficacy of VR and thus should be evaluated (55). Regarding current data, limited studies include reports of vital signs during VR distraction, and the effects were mixed. Therefore, further validation is required to obtain more objective data. Owing to the lack of objective quantifiers of pain and anxiety levels for VR distraction interventions, further in-depth studies are required to provide more accurate quantifiers for future preuse assessments.

### 4.3 Relevance to clinical practice

Overall, 22 studies demonstrated that VR use is effective in reducing pain and anxiety during the perioperative period of dental treatment. Thus, VR is effective in mitigating dental fear among patients. Furthermore, VR interventions for children at an early stage provide a good basis for future visits to promote psychological wellbeing. Patients and families are more willing to accept non-pharmacological interventions, reflecting increased patient satisfaction. Moreover, various studies have shown that VR distraction is associated with reduced stress levels in many participants (50). Using VR exclusively, or as a supplement to pharmacological analgesia, can reduce the additional costs and physical side effects associated with medication. VR distraction uses a non-invasive approach, thus mitigating concerns among parents or caregivers that general anesthesia may affect intelligence and learning ability (39). Studies have shown that the use of VR distraction for children during treatment reduces treatment duration and further validates increased compliance with medical care.

### 4.4 Outlook

With the widespread use of VR technology during dental treatment, its therapeutic effect in reducing pain and anxiety during the perioperative period has been confirmed. In future, VR technology could be integrated into dental applications, where a special assessment of the oral treatment procedure is performed, including the duration of treatment, whether anesthetic drugs should be used in combination, and patient factors, such as gender, age, level of pain or anxiety, and viewing and music preferences. These results can be used to develop personalized VR scenarios for optimal distraction. In the context of a fervently developing future metaverse, content creation of VR scenarios can be supported by the growing number of targeted VR scenarios emerging, and these continued improvements will aid in meeting dental patients’ specific needs. The accompanying disadvantage of motion sickness will undoubtedly continue to improve through more stationary scenes to keep patients from unnecessary movements during oral treatment, and they can be in a quiet state with their mouths open. In such settings, patients are in the supine position for the majority of use time, which differs from the viewing styles of patients undergoing other treatments. The viewing angle is also an important consideration, and the development of special scenes suitable for viewing during oral treatment can reduce the occurrence of motion sickness. Finally, this series of research and development can form a set of intelligent biofeedback mechanisms that will enable intelligent closed-loop anxiety and pain control throughout the perioperative period of oral treatment, thereby achieving optimal intervention effects.

Current research focuses on preoperative and intraoperative interventions, while research on postoperative anxiety and pain management is lacking. Further research is required to determine whether the use of VR applications during dental surgery can achieve full coverage in the perioperative period. Studies on VR with local anesthetics have provided better results, but those on VR in combination with opioids or light sedative medications have not yet been conducted. Future research could focus on combining the advantages of both non-pharmacological and pharmacological approaches in anticipation of using minimal drug dosages and simplifying routine protocols to achieve higher levels of comfort and satisfaction for patients undergoing oral treatment. More optimized, intelligent, and innovative solutions for perioperative behavioral management of pain and anxiety in dental treatment patients should be provided. Further research on virtual training and telemedicine assistance in dental treatment should also be considered, breaking through geographical and time limitations and integrating into the future metaverse to establish a new model of safe, comfortable, efficient, and intelligent dental treatment.

## 5 Conclusion

VR technology demonstrates enhanced efficacy in mitigating dental fear, thus contributing to improved medical safety in dental patients. Moreover, VR provides an innovative approach to non-pharmacological behavior management for doctors and nurses. Future in-depth studies can demonstrate the effectiveness of VR technology in reducing pain and anxiety during the perioperative treatment period. It is highly recommended that dental practices be targeted and personalized for VR, based on the different patient characteristics, to achieve optimal patient outcomes. Therefore, the correct implementation of measures and strategies to control anxiety in dental treatment for patients with dental anxiety disorder are as follows. 1. VR-assisted treatment is convenient and safe in all dental settings. 2. VR technology may cause certain adverse reactions, and it is recommended to evaluate and personalize its application before the dental procedure screening. 3. The application of dental sedation and anesthesia techniques is a very good complement, especially for preschool children (3–6 years), or special-needs adults who may require general anesthesia because of anxiety, fear, or physical avoidance and their inability to cooperate with treatment.

## Author contributions

NZ: Conceptualization, Writing – review & editing. LF: Formal analysis, Writing – original draft. JZ: Data curation, Visualization, Writing – review & editing. LR: Methodology, Writing – original draft. CZ: Investigation, Project administration, Software, Writing – original draft. JW: Methodology, Validation, Writing – original draft. CY: Writing – original draft.
