# Corrigendum: Virtual reality in managing dental pain and anxiety: a comprehensive review

**DOI:** 10.3389/fmed.2024.1372984

**Published:** 2024-03-20

**Authors:** Lin Fan, Jie Zeng, Longkuan Ran, Chao Zhang, Jing Wang, Cong Yu, Nan Zhao

**Affiliations:** ^1^Department of Anesthesiology, Stomatological Hospital of Chongqing Medical University, Chongqing, China; ^2^Chongqing Key Laboratory of Oral Diseases and Biomedical Sciences, Chongqing, China; ^3^Chongqing Municipal Key Laboratory of Oral Biomedical Engineering of Higher Education, Chongqing, China

**Keywords:** virtual reality, dental treatment, dental anxiety, pain, behavior management, distraction therapy

In the published article, there was an error in the author list, and the order of the listed authors is incorrect. “Lin Fan” is first author. The corrected author list appears below.

“Lin Fan, Jie Zeng, Longkuan Ran, Chao Zhang, Jing Wang, Cong Yu and Nan Zhao”

The authors apologize for this error and state that this does not change the scientific conclusions of the article in any way. The original article has been updated.

